# Community screening and treatment of asymptomatic carriers of *Plasmodium falciparum *with artemether-lumefantrine to reduce malaria disease burden: a modelling and simulation analysis

**DOI:** 10.1186/1475-2875-10-210

**Published:** 2011-07-29

**Authors:** Steven E Kern, Alfred B Tiono, Michael Makanga, Adama Dodji Gbadoé, Zulfiqarali Premji, Oumar Gaye, Issaka Sagara, David Ubben, Marc Cousin, Fiyinfolu Oladiran, Oliver Sander, Bernhards Ogutu

**Affiliations:** 1 Department of Pharmaceutics, University of Utah, Salt Lake City, Utah, USA; 2 Public Health Department, Centre National de Recherche et de Formation sur le Paludisme (CNRFP), Ouagadougou, Burkina Faso; 3 European and Developing Countries Clinical Trials Partnership (EDCTP), Cape Town, South Africa; 4 Department of Paediatrics, University of Lomé, Lomé, Togo; 5 Department of Parasitology and Medical Entomology, Muhimbili University College of Health Sciences, Dar es Salaam, Tanzania; 6 Faculty of Medicine, University Cheikh Anta Diop of Dakar (CAD), Dakar, Senegal; 7 Malaria Research and Training Center, University of Bamako, Bamako, Mali; 8 Medicines for Malaria Venture, Geneva, Switzerland; 9 Novartis Pharma, Basel, Switzerland; 10 Novartis South Africa, Johannesburg, South Africa; 11 Centre for Clinical Research, Kenya Medical Research Institute (KEMRI), Nairobi, Kenya; 12 Malaria Clinical Trials Alliance - INDEPTH Network, Nairobi, Kenya

## Abstract

**Background:**

Asymptomatic carriers of *Plasmodium falciparum *serve as a reservoir of parasites for malaria transmission. Identification and treatment of asymptomatic carriers within a region may reduce the parasite reservoir and influence malaria transmission in that area.

**Methods:**

Using computer simulation, this analysis explored the impact of community screening campaigns (CSC) followed by systematic treatment of *P. falciparum *asymptomatic carriers (AC) with artemether-lumefantrine (AL) on disease transmission. The model created by Okell *et al *(originally designed to explore the impact of the introduction of treatment with artemisinin-based combination therapy on malaria endemicity) was modified to represent CSC and treatment of AC with AL, with the addition of malaria vector seasonality. The age grouping, relative distribution of age in a region, and degree of heterogeneity in disease transmission were maintained. The number and frequency of CSC and their relative timing were explored in terms of their effect on malaria incidence. A sensitivity analysis was conducted to determine the factors with the greatest impact on the model predictions.

**Results:**

The simulation showed that the intervention that had the largest effect was performed in an area with high endemicity (entomological inoculation rate, EIR > 200); however, the rate of infection returned to its normal level in the subsequent year, unless the intervention was repeated. In areas with low disease burden (EIR < 10), the reduction was sustained for over three years after a single intervention. Three CSC scheduled in close succession (monthly intervals) at the start of the dry season had the greatest impact on the success of the intervention.

**Conclusions:**

Community screening and treatment of asymptomatic carriers with AL may reduce malaria transmission significantly. The initial level of disease intensity has the greatest impact on the potential magnitude and duration of malaria reduction. When combined with other interventions (e.g. long-lasting insecticide-treated nets, rapid diagnostic tests, prompt diagnosis and treatment, and, where appropriate, indoor residual spraying) the effect of this intervention can be sustained for many years, and it could become a tool to accelerate the reduction in transmission intensity to pre-elimination levels. Repeated interventions at least every other year may help to prolong the effect. The use of an effective diagnostic tool and a highly effective ACT, such as AL, is also vital. The modelling supports the evaluation of this approach in a prospective clinical trial to reduce the pool of infective vectors for malaria transmission in an area with marked seasonality.

## Background

Following the publication of the Millennium Development Goals in 2000, which called for a reversal in the progression of malaria by 2015 [1], an increased commitment to a number of treatment and prevention strategies has since yielded some highly successful results. The number of deaths attributable to malaria has significantly decreased in Rwanda, Ethiopia, Zanzibar, Equatorial Guinea, and Sao Tome and Principe [2-4], owing to combined strategies of mass distribution of insecticide-treated bed nets (ITN), an increased use of long-lasting insecticide-treated nets (LLIN), intermittent preventive treatment in pregnancy (IPT), and widespread adoption of artemisinin-based combination therapy (ACT). However, additional complementary interventions are still required to further accelerate the reduction in disease burden in areas that have already implemented a number of strategies to reduce disease transmission. The detection and treatment of asymptomatic carriers of *Plasmodium falciparum *as an innovative strategy for malaria control has been previously considered [5-8] and the latest WHO Guidelines for the Treatment of Malaria note that infectivity-reducing drug regimens will have a useful role to play in maintaining the reductions in disease transmission achieved through integrated control programs [9].

The transmission of *P. falciparum *parasites from humans to mosquitoes requires the presence of infectious gametocytes in the human peripheral blood. Asymptomatic carriers (AC) are individuals who harbour the *P. falciparum *asexual forms, with or without gametocytes, but do not present clinical symptoms of the disease. In malaria-endemic countries, a large proportion of *P. falciparum *infections are asymptomatic or sub-clinical. Microscopy-detected levels of asymptomatic carriage as high as 60% have been reported [5,7,10]. AC do not usually seek treatment for their infection, and therefore constitute a reservoir of parasites for the inoculation of newly-hatched mosquitoes that contributes to the transmission of the disease.

Detecting and treating asymptomatic carriers would be likely to produce benefits at both the individual level and at the community level. Patients with asymptomatic *P. falciparum *chronic infection, especially children, experience an increased morbidity due to anaemia, and reduced cognitive development [11]. In this situation, the use of surveillance with community screening and systematic treatment of asymptomatic cases becomes an important clinical tool. In addition, work has demonstrated the pervasive effect of malaria on a country's socioeconomic situation [12]. At a community-level, systematic treatment of AC could contribute to a reduced incidence of malaria, which would bring socioeconomic and healthcare benefits. A similar approach was published recently [13], which used a school based intervention of intermittent screening by rapid diagnostic tests (RDT) and treatment with artemether-lumefantrine (AL) to assess educational improvement in children. While this approach targeted a large proportion of the susceptible population, it would not decrease the disease reservoir present in younger children or adults.

For this intervention on AC to result in a reduction in disease transmission, detection of asymptomatic *P. falciparum *infection should be feasible, using a real time and reliable diagnostic tool. In addition, the anti-malarial therapy used should be effective against the transmissible sexual stage of the parasite. In comparison with non-artemisinin regimens, treatment with ACT, and in particular with AL, has been shown to result in lower gametocyte carriage rates and reduced infectivity of treated individuals [14,15]. The public health benefits of ACT in reducing gametocyte carriage are noted in the latest WHO Treatment Guidelines [9].

To explore the potential for the detection and treatment of AC to reduce the disease burden within a regional area, a modelling and simulation analysis was developed based on the published model by Okell *et al. *[16]. This model, originally developed to explore the impact of the introduction of ACT (after discontinuation of sulphadoxine-pyrimethamine) on malaria endemicity in Tanzania, was modified to simulate the impact of treating identified AC, and to explore the impact of other factors that may influence the optimization of the intervention.

This modelling and simulation analysis was designed to evaluate the impact of an intervention defined as periodic community screening campaigns (CSC) by RDT followed by systematic treatment of AC with AL (Coartem^®^) at the community level by showing a reduction in the number of confirmed symptomatic malaria episodes per person-year in the paediatric population (i.e. patients < 5 years of age) and overall population over a 12-month follow-up period, compared with no screening and treatment. If the reduction is validated in a prospective clinical study, public health policymakers may want to consider the potential of this intervention to make a significant contribution to the multifaceted approach of surveillance strategies being implemented by malaria control programmes across Africa.

## Methods

**Figure 1:**
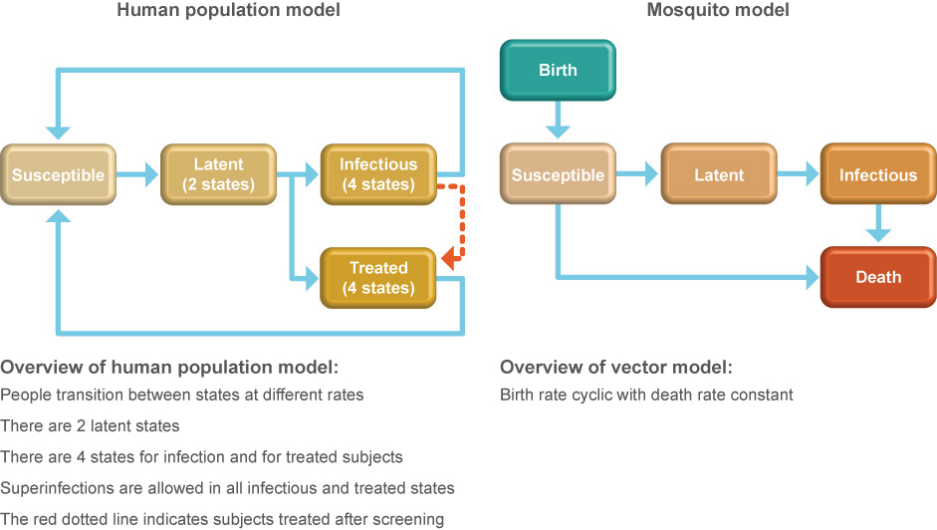
**Schematic diagram of the human and vector model states (modified from Okell *et al. *2008)**[16].

This modelling and simulation analysis utilised the basic parasite model of malaria transmission in human and mosquito populations published by Okell *et al. *[16]. This is a deterministic compartmental model that allows subjects to move between different states. Subjects initially begin in a susceptible state and become infected at a rate that is determined by mosquito density (m), the human biting rate (b), the prevalence of infectiousness in the mosquito population (A), and the probability of a subject developing a blood-stage infection. Subjects advance to two latent states before being treated or left untreated. For each of these latter two states, the model progresses through four different levels that express the degree of infectivity of the subject to mosquitoes. After this fourth stage in either state, subjects recover and return to the susceptible state. During each of the four states for treated or untreated subjects, superinfection can occur if the subject receives another infectious bite (generally with a distinct parasite strain). The rate of superinfection is set to be the same as the basic infection rate. The model was simulated using Matlab (Mathworks, Natick MA, USA). A simplified diagram of the model is shown in Figure 1.

**Figure 2:**
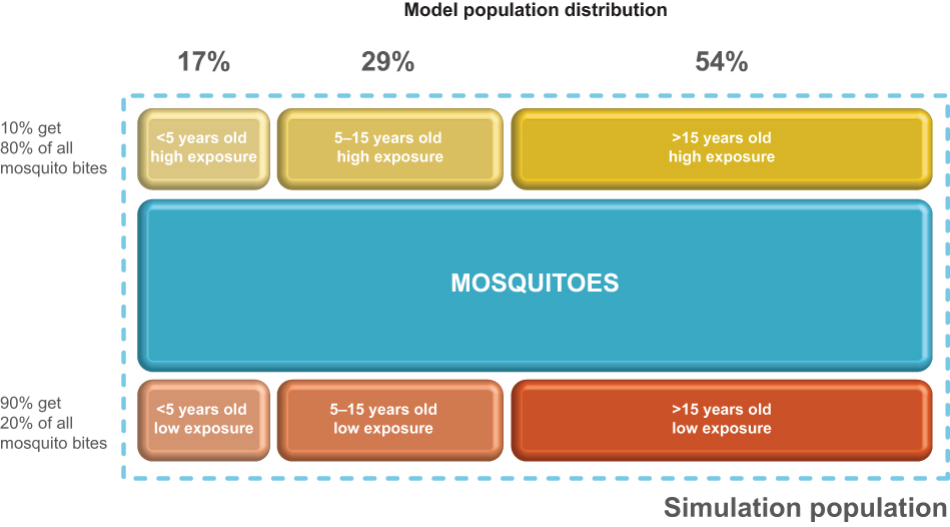
**Schematic of the population distribution based on age and propensity to receive mosquito bites**. Following the model of Okell *et al. *(2008)[16], there is heterogeneous exposure of the population to infective bites.

The subject population was divided into three groups: children aged < 5 years, children aged 5-15 years, and adolescents and adults > 15 years of age. Each of these age groups had a biting rate value, scaled according to the relative differences in body surface area and likelihood of acquired immunity, both of which increase with advancing age. The values used in the model for these parameters were those given in the original model by Okell *et al. *[16]. Additionally, there was heterogeneous exposure of the population to infective bites, and the model used the assumption that a small percentage of the population in each of these age groups (10% per group) received a large percentage of the mosquito bites (80% of bites). This group is referred to as the high biting intensity group. The remaining members of each group were subject to biting of a lower intensity and are thus referred to as the low biting intensity group (Figure 2).

A constant proportion of symptomatic subjects from each age group was treated with ACT. The effectiveness of AC detection (RDT effectiveness and population coverage) and cure rate of the ACT are parameters in the model. While the original model of Okell *et al. *[16] explored the impact of presumptive treatment of symptomatic individuals, for this analysis, the structure of the model that simulated presumptive treatment was altered to represent treatment following screening by RDT during the time of a CSC. Outside the time of a CSC, this portion of the model was disabled (dotted line in Figure 1). All simulated subjects progress through four states in either the infectious or treated branches of the model. The potential level of infectivity decreases as the subjects progress through each state until they return to the susceptible state again. Asymptomatic subjects who were initially untreated but then received treatment while in one of the four different infectivity states moved to a corresponding treatment state appropriate to the infectivity level. Additionally, the model of Okell *et al. *[16] included a protective state for subjects (prophylactic effect) prior to re-entering the susceptible state. This state was excluded from the model to reflect the relatively short half-life of AL as an ACT.

At the time of a CSC, AC were detected at a certain effectiveness rate specified in the model and treated. This only means that they were successfully diagnosed and entered the 'treated' state of the model, in which they could however still be subject to superinfection or not respond to treatment. The number and timing of the CSC can vary to assess different study designs. The durations of the wet and dry seasons were modelled using a sinusoidal function that alters the birth rate of mosquitoes in a model of the vector dynamics. The model assumes a six-month wet and dry season cycle during the course of one year; variations in the duration of each cycle were also tested (i.e. an eight-month wet season or a four-month wet season). The vector model also incorporated a susceptible state into which all the mosquitoes begin. If they became infected via a bite, they also entered a latent phase before progressing to an infectious state. Mosquitoes die at a constant rate so their density varies cyclically with the duration of the wet and dry seasons.

The model parameters that were independent of the different geographical region simulated by Okell *et al. *were set to the same values used by those authors [16]. The remaining parameters that were dependent on geographic region were adjusted to allow the dynamics of infection without the CSC intervention (but with normal ACT treatment) to vary around a range of disease prevalence rates. These included the three parameters that impact the disease intensity: mosquito density (m), the human biting rate (b), and the prevalence of infectiousness in the mosquito population (A).

Simulations were assessed with normal treatment of symptomatic malaria episodes with AL but without CSCs, to establish baseline levels of malaria incidence within a restricted population. It was assumed that there was low (< 10%) loss or cross-contamination of subjects from one village or region to another region. To assess the impact of the intervention, the model ran for a simulated time of two consecutive years without any intervention to allow the steady state condition to develop. The intervention with the series of CSCs was then started at the commencement of the dry season of Year 3.

The impact of different numbers of CSCs and the intervals between them on the simulated incidence of malaria, up to and including the complete year following the intervention, were assessed to determine the optimal placement of CSC so that the subsequent predicted incidence of malaria was minimized. As a quantitative indicator of the simulated intervention, the area under the curve (AUC) for the percentage of patients without malaria within the target age group of interest - in this analysis, children under five years of age - was calculated to assess the impact of the intervention in both the year of implementation and the subsequent year. The impacts of CSC total number, frequency and relative timing were assessed to determine the greatest impact on reducing malaria incidence within the target age group. A sensitivity analysis was also conducted to determine the influence of mosquito density, ACT cure rate, and the percentage of symptomatic infected inhabitants in the population on the model predictions.

## Results

**Figure 3:**
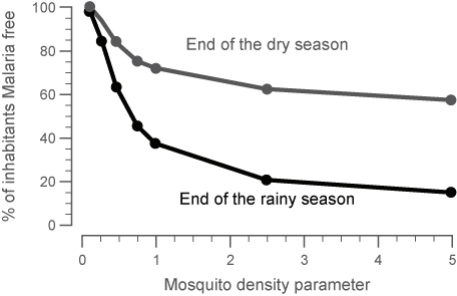
**Simulated steady state conditions that show the change in malaria incidence with the change in mosquito density within the target population of children aged < 5 years old at the end of the rainy season or the end of the dry season**. With increasing mosquito density, more subjects have malaria for longer parts of the year. At the end of the rainy season, the lowest percentage of subjects is malaria-free.

Normal cyclic patterns of malaria transmission were modelled for a range of transmission intensities, which are dependent on mosquito density and other infectivity parameters (mosquito infectiousness, human biting rate, probability that an infectious bite develops into a blood stage infection). Figure 3 shows the range of malaria incidence (asymptomatic and symptomatic) that would be expected to occur in a given region as a function of the mosquito density parameter. With an increasing number of mosquitoes, the number of susceptible subjects drops as more of the population contracts malaria. The relationship between mosquito density (m) and the entomological inoculation rate (EIR), which is commonly expressed in different malaria regions, was assessed on the basis of the model output of malaria incidence in the population. A mosquito density value of 1.25 would approximate a region of moderate endemicity (EIR < 100). A density value of 5 would approximate a highly endemic region (EIR > 200) that still possesses seasonality in the malaria incidence.

**Figure 4:**
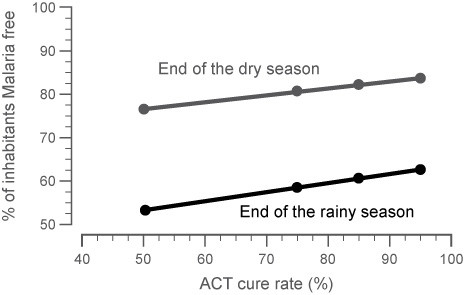
**Sensitivity analysis of the impact of ACT cure rate on the steady state malaria levels at the end of a dry or rainy season**. With increasing cure rate estimates, the number of subjects without malaria at the end of either season increases.

**Figure 5:**
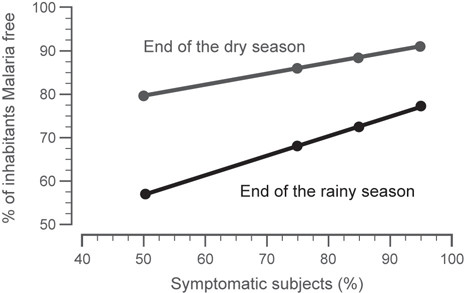
**Influence of the percentage of symptomatic subjects who have malaria on the underlying disease levels**. As more subjects are symptomatic, more are able to be treated resulting in less patients with malaria at the end of either the dry or rainy season.

A sensitivity analysis was conducted to assess the impact of model parameters on the predicted incidence of malaria within the target population. As shown in Figures 4 and 5, the impact of the treatment cure rate (the percentage of subjects cured with a standard course of AL therapy) and the percentage of inhabitants with symptomatic malaria within a population both had proportional impact on the overall rate of malaria incidence within each age group. Each model parameter was allowed to vary over a range of values to assess which had the largest effect on the model predictions. As anticipated, the parameters that had the greatest impact on the model predictions were the factors that directly influenced malaria exposure (mosquito density [m], the human biting rate [b], or the prevalence of infectiousness in the mosquito population [A]). All other model parameters had only a modest effect on the predicted percentage of patients with malaria in either the target or the whole population.

**Figure 6:**
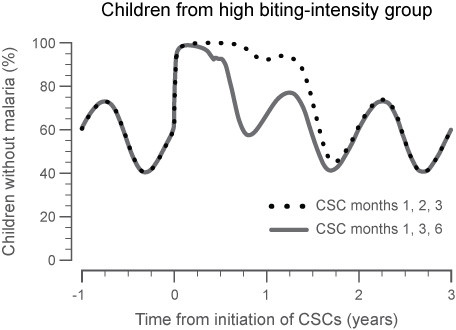
**Impact of the study treatment on parasite prevalence within a population, with moderate transmission intensity (M = 1.25, EIR < 100) and CSC scheduled at months 1, 2 and 3 or months 1, 3 and 6 of the dry season**. The panel shows the percentage of children < 5 years of age who are parasite free in the high-biting group. By repeatedly screening the population and treating the asymptomatic carriers at short time intervals, the rate of parasite prevalence can be reduced for two consecutive years as determined by the area under the curve for each year.

Conducting the entire series of CSC during the dry season and the interval between each campaign both had a large impact on the overall strategy success. The strongest decrease in malaria incidence in the target population, as indicated by the AUC for the malaria incidence, was observed with a total of three CSCs placed in close succession (separated by one month each for three months). When the CSCs were placed three months apart, at months 1, 3 and 6 of the dry season (i.e. start, middle, and end of the dry season), the effectiveness of the intervention was decreased and the subsequent impact on the next malaria season was reduced (Figure 6). CSC at months 1, 2, and 3 or at months 1, 2 and 4 of a 6-month dry season essentially showed the same overall response for reducing malaria in the following rainy season and the subsequent dry season. The addition of an extra CSC in the wet season, e.g. around the peak of transmission, did not markedly improve the success of the overall intervention. The use of a single CSC showed the least sustainable results and represents a strategy that is not likely to have a sustainable effect.

**Figure 7:**
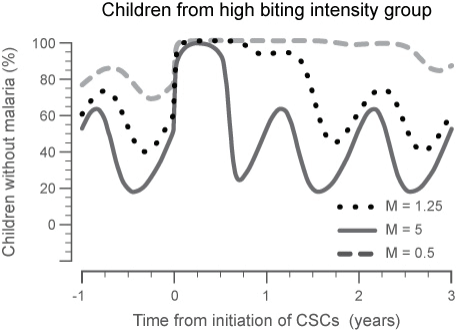
**The comparison of transmission intensity on treatment success with CSC spaced one month apart for three months**. At some level of transmission intensity (i.e. M = 5, EIR > 200), the parasite burden is too great so that eradication is not sustained within the population, indicated by the area under the curve of the malaria incidence. When the transmission intensity is low (i.e. M = 0.5, EIR < 10), the parasite burden is low and eradication is sustained within the population for at least 2 years after the intervention. This highlights the need for this strategy to be coupled with other interventions, when used in areas of moderate transmission intensity (i.e. M = 1.25, EIR < 100).

As the intensity of transmission increased, the decrease in malaria incidence within the year of intervention was still observed, but the carry-over of that reduction into subsequent years after the intervention was reduced. Thus, the sustainability of the intervention is directly related to transmission intensity in the area. Figure 7 shows that the greatest overall impact of the intervention was seen when it was implemented in an area where intensity of transmission was moderate (i.e. equivalent to EIR < 100). The results show that while malaria was reduced significantly in an area with the higher transmission intensity during the analysis year, the rate of infection returned to normal cyclic fluctuations in the immediate ensuing year, unless the intervention was repeated. Conversely, as the intensity of transmission decreased (Figure 7; M = 0.5; green curve), the impact on malaria in the region carried over for at least two years after that of the intervention. The impact of the best overall CSC strategy on an area with a low disease prevalence lasted for > 3 years after the treatment ended. This result indicates that in regions where other interventions have already reduced the malaria burden, this strategy may be an important additional factor for eliminating the remaining malaria cases within a region.

## Discussion

The results of this simulation analysis show that strategically placed community screening campaigns that are effective at treating AC of malaria will have a significant impact on reducing the disease incidence in the most vulnerable population, i.e. children below five years of age. While this population was the focus of assessment in this simulation analysis, the results showed that the incidence of malaria was reduced in the total population.

The model sensitivity analysis showed that both the efficacy of the ACT used and the percentage of AC treated had a direct linear relationship with the success of the intervention, as reflected in the minimum and maximum number of subjects without malaria within a population for a subsequent year. Thus, it is important that a highly effective therapeutic agent is used and treatment of as close to 100% of AC as possible is achieved, in order for this strategy to be effective when it is actually implemented. It is also important to have an accurate and sensitive means to detect malaria in asymptomatic patients to have a maximal impact. A conservative rate of detection and treatment of AC of 80% was chosen in this analysis. This percentage was felt to be a reasonable estimate for an aggressive screening campaign, and would take into account those subjects who might be away from their region during the timing of any of the CSC or who enter the region after cessation of the intervention (e.g. due to school attendance elsewhere, work in another region, or migration). The percentage is similar to that found by von Seidlein *et al *[17] in their study of mass drug administration to reduce malaria transmission in The Gambia. In this simulation, a conservative estimate was used for therapeutic agent efficacy of 90%, a slightly lower cure rate for AL than the > 95% efficacy typically reported in many clinical trials [18-22].

The mosquito density within a region was the factor that had the greatest impact on the success of the intervention. The lower the endemicity within a region with seasonality, the longer the effect of a single-intervention implementation would persist. At the lowest endemicity, the effect was maintained for up to five years after the implementation, as predicted by Lawpoolsri [23] in the low endemicity Thai-Myanmar border region. As the mosquito density and EIR increased in a simulated area, the ability of the intervention to reduce malaria incidence beyond the year of treatment tapered off. In order for the impact of the strategy to be sustained in a highly-endemic region, it would be best implemented as part of a strategy of combined interventions, and the intervention should be repeated with some frequency depending on the level of successful reduction achieved with other complementary approaches. This suggests that combining this approach with other effective strategies, such as LLIN universal coverage, indoor residual spraying, prompt diagnosis and effective case management, could be an important factor in achieving the goal of malaria elimination in appropriate regions.

The effect of the timing of the multiple CSC was an interesting factor that emerged from the simulation analysis. By clustering the campaigns earlier in the dry season and in relatively quick succession, the incidence of malaria in the transmission season after intervention was more effectively reduced. This is likely due to the latency period between an infectious bite and the development of blood stage parasitaemia. By rapidly repeating the CSC, subjects who were not detected in the initial campaign could be detected in the subsequent campaign and immediately treated. This prevents the disease from developing to its full stage of progression; a full cycle in the model generally takes almost 6 months to complete and return to the susceptible state again. The disease progression was modelled using ordinary differential equations that did not account for the potential to have stochastic bursts of malaria within a region or subgroup of patients. If such features of the disease were to appear, particularly in the post-intervention assessment period, it could influence the estimated success of the intervention in a region.

**Figure 8:**
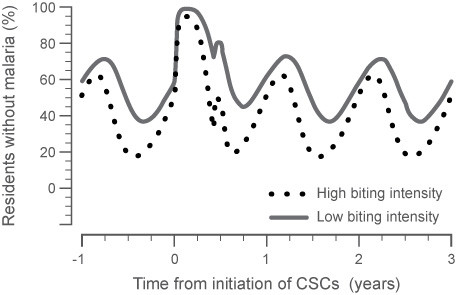
**Model prediction calibration simulating a single CSC held at the start of the dry season**. This approach is representative of the study by von Seidlein *et al *in The Gambia where a single mass screening campaign was undertaken in a high intensity region. As the study showed, the impact was not sustained and resulted in higher susceptibility for a short time immediately after the intervention (irrespective of the group considered, high or low-biting intensity).

In the study by von Seidlein *et al. *in The Gambia [17], there was only a single treatment campaign of 85% of the population without prior screening. The treatment campaign used artesunate-sulphadoxine-pyrimethamine, a therapeutic agent that had lower reported efficacy than AL for clearing mature gametocytes in asymptomatic subjects. In addition, a number of villages from the treated group and the placebo group were often only 2 km apart. Consequently, it is unlikely that the 'treated' and 'control' human and vector populations were clearly separated from one another. All these factors may have contributed to the lack of effect on malaria incidence found in this study, as confirmed in a simulation of this study approach with the model presented here. Figure 8 shows a good representation of the outcome of von Seidlein [17], which emphasizes the importance of active screening in multiple successive CSC. The von Seidlein *et al. *study was also undertaken in a highly endemic region prior to the widespread utilization of other interventions such as LLIN. As the present simulation analysis shows, the relative level of disease intensity has a large impact on how successful a single treatment cycle of asymptomatic patients is likely to be. Reducing the pool of infectious vectors in this population is only a partial step in disease eradication if it is not combined with other effective strategies.

The results of this study complement the recent publication by Griffin *et al. *[24] that also used the Okell model to evaluate malaria intervention strategies across different malaria transmission settings found in Africa. Griffin and colleagues found that with increasing transmission intensity, reflected by a higher EIR, combinations of intervention are necessary to reduce malaria transmission levels significantly. For regions with moderate and high transmission intensity, systematic screening and treatment paradigms would reduce parasite prevalence in a region, but would have to be repeated for sustained effect. In a complementary manner, the results provide an estimate of the frequency and characteristics of this type of intervention that would be necessary for sustaining transmission reduction.

The addition of seasonality to the Okell model allows for the assessment of the dynamics of malaria transmission. The extended period of time between when a subject is first infected and exhibits clinical symptoms adds to the complexity of treating this disease. Taking a systems engineering viewpoint to the question of when to best place the CSCs to control disease spread, it becomes apparent that the placement and interval between the campaigns has to harmonize with the natural frequency of the disease spread. In this regard, strategically placed CSC should complement other interventions, such as IPT in pregnancy, the deployment of LLINs and programmes of indoor residual spraying.

## Conclusions

In conclusion, this simulation analysis identified conditions that may contribute to the successful implementation of an intervention to reduce the pool of infectious vectors for malaria transmission by treating AC of *P. falciparum*. By doing so in a region with marked seasonal transmission and a moderate malaria incidence such as the Sahelian zone (Senegal, Mali, Burkina Faso), and by combining this with other successful interventions, there is an opportunity to reduce malaria transmission, particularly in those most at risk. It is likely that combining this approach with already existing methods for vector control will have a more lasting impact, particularly in regions with high disease incidence.

This simulation analysis highlights the utility of relatively simple models of malaria disease dynamics and therapeutic impact that may be important for planning a potentially complicated and resource-intense clinical study to further explore this treatment strategy. The benefit of malaria modelling was noted in a perspective article published in parallel with the original Okell modelling paper [25]. This article concludes that mathematical modelling may help to preserve the efficacy of currently available ACT, monitor for the emergence of resistance, and contribute to malaria control. The approach described in this analysis should allow an upcoming clinical trial in Burkina Faso to prospectively assess the potential for the treatment of AC to make a contribution to the multifaceted approaches currently being utilized across Africa. As malaria elimination becomes a realistic probability in certain epidemiological settings, the treatment of AC could work in conjunction with existing interventions such as prompt diagnosis by RDT and effective case management, IPT, and vector control to build upon the achievements of the past 10 years. The results of this simulation support the testing of the hypothesis in a clinical study to assess its validity.

## List of abbreviations

ACT: artemisinin-based combination therapy; AL: artemether-lumefantrine; AUC: area under the curve; CSC: community screening campaign; EIR: entomological inoculation rate; IPT: intermittent preventive treatment; ITN: insecticide-treated nets; LLIN: long-lasting insecticide-treated nets; m: mosquito density; RDT: rapid diagnostic test.

## Competing interests

DU is an employee of Medicines for Malaria Venture. SK, MC and OS are employees of Novartis Pharma AG, the manufacturer of artemether-lumefantrine (Coartem^®^). MC holds stock with Novartis. FO is an employee of Novartis South Africa, a subsidiary of Novartis Pharma AG, the manufacturer of artemether-lumefantrine (Coartem^®^). The other authors declare no competing interests.

## Authors' contributions

All authors met International Committee of Medical Journal Editors criteria for authorship. All authors revised the manuscript critically for important intellectual content, and read and approved the final manuscript.
